# Clinical impact of PD-L1 expression in triple-negative breast cancer patients with residual tumor burden after neoadjuvant chemotherapy

**DOI:** 10.1186/s12957-021-02361-9

**Published:** 2021-09-02

**Authors:** Gizem Oner, Semen Önder, Hüseyin Karatay, Naziye Ak, Mustafa Tükenmez, Mahmut Müslümanoğlu, Abdullah İğci, Ahmet Dincçağ, Vahit Özmen, Adnan Aydiner, Ekrem Yavuz, Neslihan Cabioğlu

**Affiliations:** 1 Department of General Surgery, Istanbul Faculty of Medicine, Istanbul University, Istanbul, Turkey; 2 Multidisciplinary Oncologic Centre Antwerp (MOCA), Antwerp University Hospital, Edegem, Belgium; 3 Center for Oncological Research (CORE), University of Antwerp, Wilrijk, Belgium; 4 Department of Pathology, Istanbul Faculty of Medicine, Istanbul University, Istanbul, Turkey; 5 Department of Medical Oncology, Institute of Oncology, Istanbul University, Istanbul, Turkey

**Keywords:** Triple-negative breast cancer, PD-L1 expression, Prognosis, Neoadjuvant chemotherapy response

## Abstract

**Background:**

Studies on PD-L1 expression in breast cancer have gained importance in recent years, especially in triple-negative breast cancer (TNBC). Our aim was to analyze the differential expression of PD-L1 to explore its correlation with response to neoadjuvant chemotherapy (NACT) and patient survival.

**Methods:**

PD-L1 expression was evaluated immunohistochemically (Ventana SP263 clone kit) by staining tumor specimen. PD-L1 positivity was defined as membranous staining > 1%, > 5%, > 10%, and > 20% on either tumor cell (TC) and /or immune cell (IC).

**Results:**

Fifty patients with locally advanced TNBC, who had a partial response to NACT, were included in the study. PD-L1 staining was observed in TCs in 25 patients (50%) and in ICs in 23 patients (46%) when PD-L1 > 1% was considered positive. Patients with PD-L1 positivity on ICs were more likely to respond to chemotherapy as measured by “MD Anderson Cancer Center Residual Cancer Burden Index” (14/22, 63.6% vs. 10/27, 37%, *p* = 0.064). The 5-year disease-free survival (DFS) and disease-specific survival (DSS) rates were 46.3% and 51.4%, respectively. A high (> 20%) tumoral PD-L1 positivity was associated with a better DFS and DSS.

**Conclusions:**

Studies in the literature mostly focused on PD-L1 expression in inflammatory cells. However, our results suggest that patients with a high PD-L1 expression on TCs were more likely to have a better outcome. Since patients with residual tumor burden who express PD-L1 on TILs were more likely to respond to NACT, an immune checkpoint inhibitor therapy in addition to NACT would be an important option for TNBC with locally advanced disease.

## Introduction

Triple-negative breast cancer (TNBC) is a heterogeneous type of breast cancer that is characterized by the absence of expression of estrogen receptor (ER), progesterone receptor (PR), and human epidermal growth factor receptor-2/neu (HER-2). TNBC has a high degree of aggressiveness, and generally has a worse prognosis than other types of breast cancer [1, 2]. TNBC still lacks targeted treatment options; therefore, chemotherapy remains the main treatment method. The use of neoadjuvant chemotherapy (NAC) is the standard of care in TNBC, including early stage. Patient who has a pathologic complete response (pCR) with NAC is characterized wıth improved survival outcome [3–5].

The complex role of the immune system in breast cancer growth, elimination, and metastasis has been the object of increased attention especially in TNBC. Recent evidence highlights the pivotal role of immune checkpoint receptors in TNBC. On the other hand, there are no approved targeted therapies for TNBC in the neoadjuvant setting. Early results from clinical trials with inhibitors of this pathway have validated its potential as a target for cancer immunotherapy. PD-1 is an important immune checkpoint molecule, which together with its principal ligand PD-L1 plays an essential role in the clinics for TNBC [6, 7]. Tumors can escape antitumor immune activity by exploiting upregulated PD-L1 expression in the tumor microenvironment [8, 9].

Although considerable research has been devoted to PD-L1 expression level in TNBC, less attention has been paid to PD-L1 prognostic value in survival. This paper attempts to shed light on PD-L1 expression in neoadjuvant treatment TNBC and its correlation with clinical outcome.

## Material and methods

Between 2002 and 2018, 853 patients diagnosed with locally advanced breast cancer received neoadjuvant chemotherapy (NAC) at Istanbul University, Istanbul Faculty of Medicine, Department of General Surgery. Of those, 50 consecutive patients who were diagnosed with TNBC without distant metastases, and had undergone surgery following NAC were included in this study. Patients who did not complete NAC and did not have follow-up less than 24 months, as well as patients with pathological complete response after neoadjuvant therapy were excluded from the study. Two patients with inflammatory breast cancer and 6 patients with metaplastic breast cancer were included. Demographic characteristics, tumor characteristics, and follow-up time were analyzed retrospectively.

Estrogen and progesterone receptors and c-erb-B2 were examined immunohistochemically (IHC). Expressions < 1% for estrogen receptors (ER) and progesterone receptors (PR) were considered negative. Immunohistochemical evaluation of c-erb-B2 was performed according to the percentage of staining of the invasive carcinoma cells and the staining quality (weak-medium-strong/incomplete-complete membrane) and in accordance with the suggestions by American Society of Clinical Oncology/College of American Pathologists (ASCO/CAP). Immunostaining score of 0 is considered negative, while scores 1+ and 2+ were confirmed by fluorescence in-situ hybridization (FISH) or by chromogenic in-situ hybridization (CISH). The determination of these markers has been a standard part of the pathology report at our hospital. For this reason, patients with TNBC were selected based on the results of the previous pathology reports.

Tumor paraffin block sections with excess lymphocyte expression were selected. PD-L1 expression was detected by using “Rabbit monoclonal antibody, Ventana SP263 Clone kit” with an automatic device (VENTANA BenchMark automatic slide staining device). A placenta tissue was used as a control group.

### Immunohistochemical evaluation and scoring

Positive staining rates (×400, HPF) of tumor cell (TC), and/or immune cell (IC) were evaluated under the light microscope*.* Membranous staining %≥ 1 on TCs and/or ICs was considered positive for PD-L1, while %≥ 5 and %≥ 10 and %≥ 20 stainings were considered as high PD-L1 expression.

“MD Anderson Cancer Center Residual Cancer Burden Index” was used to measure chemotherapy response. The following parameters are required in order to calculate residual cancer burden (RCB) after neoadjuvant treatment:

- The two largest dimensions of the residual tumor bed (the largest tumor bed in multi-centric cases is included in the calculation)
- The histologic assessment of the percentage of the tumor bed area that contains carcinoma
- The histologic estimate of the percentage of the carcinoma in the tumor bed that is in-situ
- The number of metastatic lymph nodes
- The diameter of the largest lymph node metastasis

These variables were loaded to the MD Anderson Residual Cancer Calculator (www3.mdanderson.org/app/medcalc/index.cfm?pagename = jsconvert3), and then “RCB” was obtained, and the residual cancer classification was made according to this scoring. In this classification, 0 = is associated with pathological complete response, whereas 3 = is considered as chemotherapy resistant

### Statistical analysis

The statistical analysis of the study was performed by using the statistical software program SPSS 17 (Statistical Package for Social Sciences; SPSS, Inc, Chicago, IL). A *p* value less than 0.05 was considered statistically significant. Categorical variables were evaluated by Fisher’s exact test. Disease-free survival rates were analyzed by considering local and systemic metastases, and disease-specific survival rates were analyzed considering breast cancer-related mortality. Kaplan-Meier analyses were used for the survival curves test also known as Mantel-Cox test log rank test, and log rank test was used to compare factors affecting outcome.

## Results

### Clinical and pathological findings

Median age was 47.5 (24–76) years. The demographic and pathological characteristics of the patients are shown in Table 1. When clinically evaluated before neoadjuvant chemotherapy three patients were T1 (6%), 18 patients were T2 (36%), 7 patients were T3 (14%), and 22 patients were T4 (44 %). All of these patients received anthracycline and taxane chemotherapy protocols, and three patients (6%) received additional platinum chemotherapy regimen. Two of these patients (4%) were N0 before neoadjuvant chemotherapy, 30 of them were N1 (60 %), 11 patients (22%) were N2, and 14% (*n* = 7) of the total were clinically N3. Only one patient was known to have bone metastasis before neoadjuvant chemotherapy. Following the neoadjuvant chemotherapy, modified radical mastectomy was performed on the majority of patients (62%) (Table 1). Twenty-two patients were pT1 (44%), 16 patients (32%) were pT2, 9 patients (18%) were pT3, and 3 patients (6%) were pT4. Furthermore, 17 (34%) patients had pathological complete response in axillary lymph nodes diagnosed with pN0, 14 patients (28%) with pN1, 9 patients (18%) with pN2, and 10 patients (20%) with pN3 following NAC. In 82.3% of the patients, Ki-67 score was > %20, and 72.2% of patients had Ki-67 score > 35%.

**Table 1:** Demographic and pathological features of patients

Patients characteristics	***N*** = 50 (%)
Median age	47.5 (min-maks; 24–76)
Premenopausal	23 (46%)
Postmenopausal	27 (54%)
Family history	
Yes	6 (12%)
No	44 (88%)
Clinic T	
T1	3 (6%)
T2	18 (36%)
T3	7 (14%)
T4	22 (44 %)
Clinic N	
N0	2 (4 %)
N1	30 (60 %)
N2	11 (22 %)
N3	7 (14 %)
Pathological T	
T1	22 (44%)
T2	16 (32%)
T3	9 (18 %)
T4	3 (6 %)
Pathological N	
N0	17 (34%)
N1	14 (28%)
N2	9 (18%)
N3	10 (20%)
Surgical procedures	
Modified radical mastectomy	31 (62%)
Mastectomy and SLNB (+) and ALND	5 (10%)
Mastectomy and SLNB (−)	3 (6%)
BCS &ALND	3 (6%)
BCS &SLNB (+) and ALND	4 (8%)
BCS& SLNB (−)	4 (8%)
Pathological findings	
Invasive ductal carcinoma	39 (78%)
Invasive lobular carcinoma	3 (6%)
Metaplastic	6 (12%)
Invasive ductal carcinoma+ invasive lobular carcinoma	1 (2%)
Undifferentiated carcinoma	1 (2%)

*BCS* breast conserving surgery, *SLNB* sentinel lymph node biopsy, *ALND* axillary lymph node dissection

Since one patient had a residual tumor tissue only in the lymphovascular area, the residual cancer evaluation could not be done for that patient. The response of patients to chemotherapy was evaluated by the “MD Anderson Cancer Center Residual Cancer Burden Index.” The median score was 3.49 (0.72–5.07) and chemotherapy response was worse in 27 patients (55.1%) (class III). The chemotherapy response was moderate in 20 patients (40.8%, class II), while 2 patients (4.1%, class I) responded well to chemotherapy (Table 2).

**Table 2:** MD Anderson Cancer Center Residual Cancer Burden Index

Residual Cancer Score	*N* = 49*
Class I	2 (4.1%)
Class II	20 (40.8%)
Class III	27 (55.1%)

*The MD Anderson Cancer Center Residual Cancer Burden Index cannot be calculated in one patient with tumor cells present only in the lymphovascular space

### Immunohistochemical staining findings

**Fig. 1:**
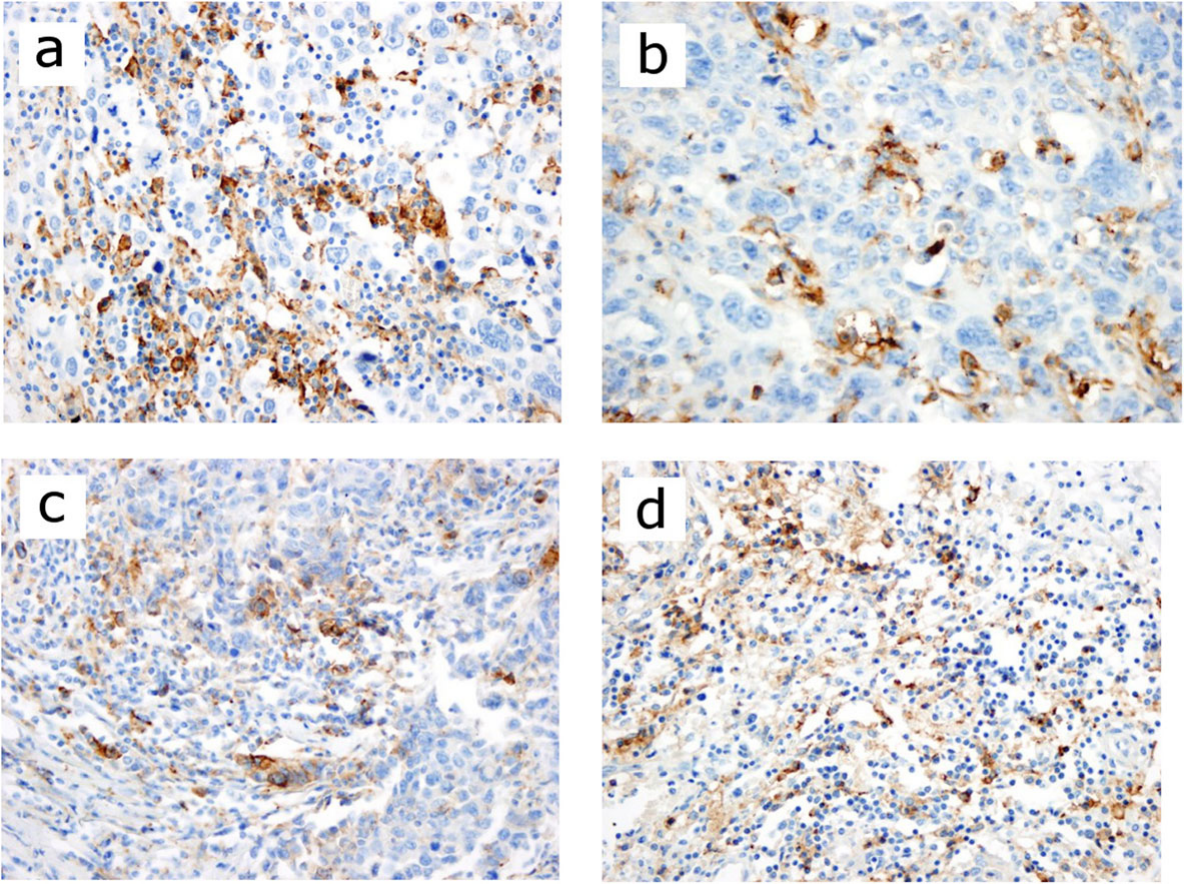
**a** PD-L1 immunohistochemical expression is 20% on the tumor and 10% on the TILs (×400, HPF). **b** PD-L1 immunohistochemical expression is 25% on the tumor and 20% on the TILs (×400, HPF). **c** PD-L1 immunohistochemical expression is 15% on the tumor and 20% on the TILs (×400, HPF). **d** 1-PD-L1 immunohistochemical expression on the tumor 2% and 10% on the TILs (×400, HPF)

When PD-L1 > 1% was considered positive, PD-L1 staining has been observed on TCs in 25 patients (50%) and on ICs in 23 patients (46%) (Fig. 1). PD-L1 positivity on TCs and/or ICs was seen in 26 patients (52%) (Table 3). PD-L1 > 5% positivity was detected on TCs in 16 patients (32%) and on the ICs in 21 patients (42%). In addition, PD-L1 > 10% positivity was found on TCs in 13 patients (26%) and on the ICs in 15 patients (30%). Furthermore, PD-L1 > 20% positivity was considered to be high expression, which was detected on TCs in 7 patients (14%) and on the ICs in 6 patients (12%).

**Table 3:** PD-L1 staining patterns and neoadjuvant chemotherapy response in TNBC patients

PD-L1 staining	***N*** = 50	Class I and II (***n*** = 22)	Class III (***n*** = 27)	***P*** value
Tumoral PD-L1 ≥ %1	25 (50 %)	11	14	0.999
TILs PD-L1 ≥ %1	23 (46 %)	14	10	0.064*
Tumoral and/or TILs PD-L1 ≥ %1	29 (58%)	14	15	0.771
Tumoral PD-L1 ≥ %5	16	8	8	0.761
TILs PD-L1 ≥ %5	21	11	10	0.398
Tumoral and/or TILs PD-L1 ≥ %5	23	12	11	0.396
Tumoral PD-L1 ≥ %10	13	7	6	0.525
TILs PD-L1 ≥ %10	15	8	7	0.538
Tumoral and/or TILs PD-L1 ≥ %10	19	10	9	0.556
Tumoral PD-L1 ≥ %20	7	4	3	0.685
TILs PD-L1 ≥ %20	6	2	4	0.678
Tumoral and/or TILs PD-L1 ≥ %20	9	4	5	0.999

Tumor Infiltrating (stromal) lymphocytes (=TILs)

PD-L1 expression was found in all of the patients with inflammatory breast cancer (*n* = 2) and in 5 of 6 patients with metaplastic breast cancer. In Pearson correlation analysis, PD-L1 expression on ICs and TCs correlated with high significance (*p* = 0.0001, Pearson correlation 0.550). According to Residual Cancer Index, good/medium responders (*n* = 22) and bad responders (*n* = 27) were analyzed with Fisher test in relationship to positivity of PD-L1 on TCs or ICs. It was seen that PD-L1 positivity was highly expressed on ICs (14/22, 63.6% vs. 10/27, 37%, *p* = 0.064) in the group that responded better to chemotherapy. However, this did not reach statistical significance level. There was no statistical significance between PD-L1 expression on TCs and/or ICs and chemotherapy response.

**Fig. 2:**
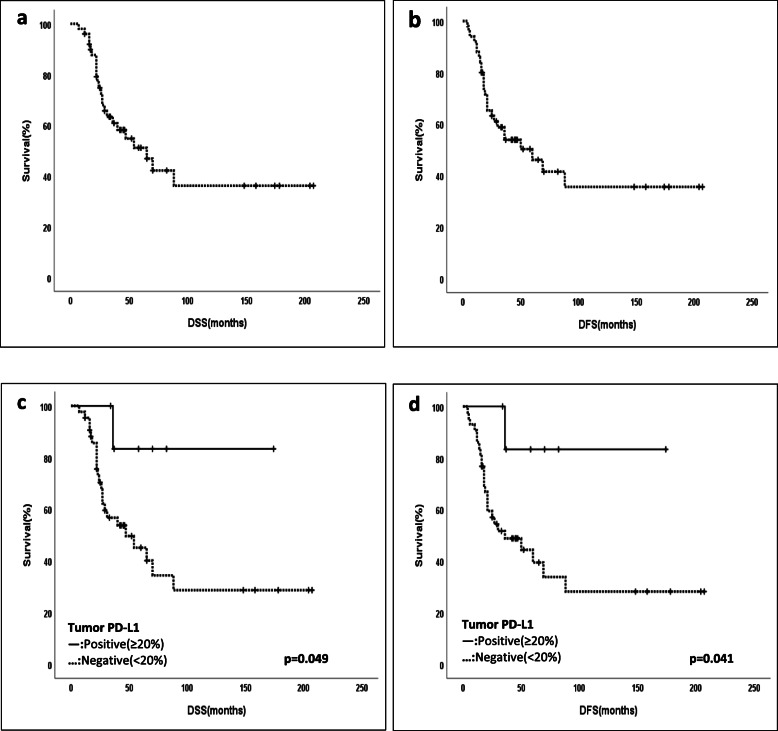
**a** 5-year disease-specific survival (DSS). **b** 5-year disease-free survival (DFS). **c** High tumor PD-L1 (+) (> 20%) expression (positive = 1, negative = 0) and disease-specific survival. **d** High tumor PD-L1 (+) (> 20%) expression (positive = 1, negative = 0) and disease-free survival

The median follow-up time was 35 months (7-207). The 5-year DFS and DSS were 46.3% and 51.4% for the whole cohort, respectively. In Kaplan-Meier analysis, patients with > 20% tumoral PD-L1 expressions had a better 5-year disease specific survival rate (DSS) and better 5-year disease-free survival rate (DFS). It was a statistical significance (DFS; *p* = 0.041 and DSS *p* = 0.049) (Fig. 2). Furthermore, the other associations regarding different PD-L1 expression and DFS or DSS were not found statistically significant (Table 4).

**Table 4:** Outcome of patients according PD-L1 expression patterns

*N* = 50	5-Year disease-free survival (DFS)	*P* value	5-Year disease-specific survival (DSS)	*P* value
Tumor PD-L1 (+) (≥ 1%)	47.7%	0.90	51.4%	0.877
Tumor PD-L1 (−)	44.6%		50.9%	
Tumor PD-L1(+) (≥ 5%)	54.7%	0.55	61.1%	0.258
Tumor PD-L1(−)	40.9%		44.4%	
Tumor PD-L1 (+) (≥ 10%)	67.3	0.095	67.3%	0.085
Tumor PD-L1 (−)	37.5		44.2%	
Tumor PD-L1 (+) (≥ 20%)	83.3%	**0.041**	83.3%	**0.049**
Tumor PD-L1 (−)	39.8%		45.1%	
Lymphocyte PD-L1 (+) (≥ 1%)	56.6%	0.471	60.0%	0.292
Lymphocyte PD-L1 (−)	37.7%		42.7%	
Lymphocyte PD-L1 (+) (≥ 5%)	60.6%	0.310	60.3%	0.323
Lymphocyte PD-L1 (−)	35.8%		44.7%	
Lymphocyte PD-L1 (+) (≥ 10%)	65.2%	0.263	65.2%	0.277
Lymphocyte PD-L1 (−)	38.7%		45.6%	
Lymphocyte PD-L1 (+) (≥ 20%)	66.7%	0.349	66.7%	0.249
Lymphocyte PD-L1 (−)	42.5%		48.6%	
Tumor and lymphocyte PD-L1 (+) (≥ 1%)	47.9%	0.959	51.1%	0.838
Tumor and lymphocyte PD-L1 (−)	43.8%		51.6%	
High tumor and lymphocyte PD-L1 (+) (≥ 5%)	59.8%	0.260	64.0%	0.108
High tumor and lymphocyte PD-L1 (−)	33.7%		38.6%	
High tumor and lymphocyte PD-L1 (+) (≥ 10%)	67.0%	0.098	67.0%	0.119
High tumor and lymphocyte PD-L1 (−)	33.5%		41.3%	
High tumor and lymphocyte PD-L1 (+) (≥ 20%)	66.7%	0.188	66.7%	0.144
High tumor and lymphocyte PD-L1 (−)	40.5%		46.8%	

## Discussion

PD-L1 is expressed on the different cell types, including TCs and ICs, and the presence of PD-L1 in the tumor microenvironment seems to indicate an immune resistance to endogenous antitumor activity [10, 11]. The studies on PD-L1 expression in breast cancer have gained importance in recent years. In these studies, different rates of PD-L1 expression are seen in each of the breast cancer subgroups. For this reason, the frequency of PD-L1 expression varies in studies [12–14]. The prognostic and predictive values of PD-L1 in published studies are also controversial [12–18]. Different results in publications are due to the different methods to determine PD-L1 expression (determination of mRNA expression by IHC expression, using paraffin tissue blocks, using tissue microarray, different monoclonal kits used in IHC staining) and the differences in scoring systems. Gonzalez-Ericsson et al reported that results on TNBC showed discrepancies between SP142, SP263, and 22C3 assays. SP142 has a lower PD-L1 expression on both TC and IC compared to other assays [19]. Moreover, some drug studies also have begun to use combined positive score (CPS), which is the number of PD-L1 staining cells (tumor cells, lymphocytes, macrophages) divided by the total number of viable tumor cells, multiplied by 100 [20]. In the study by Soliman et al. with flow cytometry on breast cancer subgroups, PD-L1 expression was shown to be greater in the basal-type cancer group than in the luminal group [21]. Ghebeh et al. demonstrated in their studies that PD-L1 expression is associated with the tumor characteristics such as a high grade, estrogen receptor negativity and an increased T-regulatory (T-reg) expression [22, 23]. In the study of Morgan et al., it was shown that PD-L1 is expressed in tumor cells in medullary type breast cancer more than TNBC [24]. The first study that investigated PD-L1 expression (defined as cell-surface membrane staining > 5%) in breast cancer found a higher PD-L1 expression in TNBCs as compared to non-TNBCs (*p* < 0.001) [13]. Furthermore, intratumoral CD8^+^ T cells were more likely to be found in the PD-L1-positive group compared to the others [13]. According to the results of a study of Li et al., PD-L1 was more likely to be expressed on immune cells in regards to tumor cells and the prevalence of PD-L1 was found to express in similar rates on primary and metastatic TNBC samples [25]. Our study was carried out in the locally advanced TNBC patients who received neoadjuvant chemotherapy. Because different PD-L1 scoring systems are used in literature, we decided to utilize different cut-off values for PD-L1 expression. It is critical to appreciate the true impact of the PD-L1 expression level in TME so that PD-L1 positivity was defined as any membranous staining ≥ %1, whereas ≥ %5 and ≥ %10 and ≥ %20 staining were considered as high PD-L1 positivity. The correlation between PD-L1 levels and inhibition of anticancer immunity is currently unknown and also different level of PD-L1 expression might have different significant biological consequences. Beckers et al. firstly pointed out that PD-L1 was also expressed on TILs in breast cancer [26]. Our study also confirmed that the percentage of PD-L1 expression on lymphocyte and tumor was highly correlated (*p* = 0.0001).

Bianchini et al. stated in their study that c-erb-B2-positive patients with an increased expression of PD-L1 had impaired immunological control mechanisms, resulting in poor response to neoadjuvant chemotherapy [17]. In another study, it was shown that the patients with high expression of PD-L1 was associated with a higher rate of pathologically complete response rate compared to the other group (50% vs. 21%) [12]. In this study, the patients were mostly chemotherapy-resistant and the chemotherapy response in this patient group was assessed by the “MD Anderson Cancer Center Residue Cancer Burden Index Neoadjuvant chemotherapy response.” The analytical results of this study supported the view that the PD-L1 expression on ICs correlated with better response to chemotherapy. (14/22, 63.6% vs. 10/27, 37%, *p* = 0.064).

There are also controversial results in published studies regarding the prognostic effect of PD-L1 expression. In the study by Muenst et al., patients with increased PD-L1 expression were found to have a poor prognosis [8]. Contrarily, Schalper et al. showed that patients with high PD-L1 expression on the ICs had a better prognosis [18]. In our study, there was a significant difference between 5-year DFS rates and DSS rates among the patients with ≥ %20 tumoral strong staining PD-L1 positivity and PD-L1 negativity. In other words, high PDL-1 expression on TCs was associated with longer survival rate, and this result shows that PDL-1 expression on TCs may be more important than expected as a predictive and prognostic marker. In addition, first findings of Keynote-119 study have shown that pembrolizumab monotherapy versus chemotherapy did not significantly increased overall survival (OS) in metastatic TNBC. On the other hand, median OS was 14.9 months with pembrolizumab versus 12.5 months with chemotherapy (Hazard ratio [HR], 0.58; 95% CI, 0.38–0.88) in patients with a Combined Positive Score (CPS) ≥ 20 [20].

PD-1/PD-L1 inhibitory treatment in neoadjuvant setting is becoming more important [27, 28]. PD-L1 expression on ICs is also associated with clinical benefit from PD-1/PD-L1 inhibitors therapy, as demonstrated in both non-small cell lung cancer and urothelial cancer [29, 30]. Currently, several large randomized studies showed that PD-1/PD-L1 inhibitors in combination with neoadjuvant chemotherapy for advanced TNBC breast cancer were associated with an important clinical benefit [31, 32]. In the I-SPY-2 trial, paclitaxel was administered with or without pembrolizumab, followed by doxorubicin with cyclophosphamide in women with locally advanced HER2-disease [31]. The estimated pCR was approximately 20% in the control arm versus 60% in the arm containing pembrolizumab for the subcategory of women with TNBC. The phase III IMpassion 130 trial enrolled 902 patients with metastatic TNBC who had not received prior treatment for metastatic disease [32]. Patients were randomly selected to standard chemotherapy (nab-paclitaxel) plus atezolizumab, a PD-L1 inhibitor, or to standard chemotherapy plus placebo. A clinical benefit with atezolizumab-nab-paclitaxel was particularly notable in the PD-L1-positive group. Objective response rate was higher with the combination compared to chemotherapy alone for all patients (56% vs. 46%) and those with PD-L1-positive tumors (58.9% vs. 42.6%). Although differences in OS between patients receiving atezolizumab and those not receiving atezolizumab were not statistically significant in the IMpassion130 trial, clinically significant OS benefit was observed in those who were PD-L1-positive (mean OS improvement of 7.5 months) [33]. The KEYNOTE-173 study showed that PD-L1 combined positive score (CPS) and stromal TIL levels were strongly correlated with each other [34]. For this reason, it was not clear whether they are independent predictors or prognostic factors. In the GeparNuevo study, PD-L1 expression on TCs with SP263 predicted the response to durvalumab in the neoadjuvant setting [35].

Furthermore, combination immunotherapy studies have shown better response compared to monotherapies using PD-1/PD-L1 inhibitors in cancer treatment [36]. Therefore, novel biomarkers associated with response to chemotherapy and prognosis should also be investigated to enhance the chemotherapy response and improve the outcome in TNBC [37–39].

## Conclusion

It is widely accepted that PD-L1 is highly expressed in TNBC. On the other hand, there are different findings in the literature about the predictive and prognostic value of PD-L1 expression regarding its level and expression pattern. PD-L1 expression on the ICs may be indicative for a better prognosis enabling, with a higher response rate to chemotherapy, whereas high PD-L1 expression on TCs may be more associated with DFS and DSS. However, questions regarding which PD-L1 expression levels are more significant or whether PD-L1 expression on ICs or TCs is more predictive and prognostic are to be answered. In the future, PD-1/PD-L1 inhibition will also be an alternative adjuvant treatment option for TNBC patients with the residual tumor burden after neoadjuvant chemotherapy but further investigations are necessary to improve our understanding of PD-L1.

## Data Availability

The datasets during and/or analyzed during the current study available from the corresponding author on reasonable request.
