# Correction: Clinical impact of PD-L1 expression in triple-negative breast cancer patients with residual tumor burden after neoadjuvant chemotherapy

**DOI:** 10.1186/s12957-023-02931-z

**Published:** 2023-02-21

**Authors:** Gizem Oner, Semen Önder, Hüseyin Karatay, Naziye Ak, Mustafa Tükenmez, Mahmut Müslümanoğlu, Abdullah İğci, Ahmet Dincçağ, Vahit Özmen, Adnan Aydiner, Ekrem Yavuz, Neslihan Cabioğlu

**Affiliations:** 1 Department of General Surgery, Istanbul Faculty of Medicine, Istanbul University, Istanbul, Turkey; 2 Multidisciplinary Oncologic Centre Antwerp (MOCA), Antwerp University Hospital, Edegem, Belgium; 3 Center for Oncological Research (CORE), University of Antwerp, Wilrijk, Belgium; 4 Department of Pathology, Istanbul Faculty of Medicine, Istanbul University, Istanbul, Turkey; 5 Department of Medical Oncology, Institute of Oncology, Istanbul University, Istanbul, Turkey

**Correction: World J Surg Onc 19, 264 (2021)**

**https://doi.org/10.1186/s12957-021-02361-9**

Following publication of the original article [1], the author reported an error in the “Funding“ section.

“This Project was supported by the Istanbul University, Department of Scientific Research Projects (ID26409/TTU-2017-26409), and the Istanbul Breast Society. “

Should be:

This Project was supported by the Istanbul University, Department of Scientific Research Projects (ID26409/TTU-2017-26409), **(ID27023/TOA-2017-27023)** and the Istanbul Breast Society.

The original article has been updated.
